# The HDL from septic-ARDS patients with composition changes exacerbates pulmonary endothelial dysfunction and acute lung injury induced by cecal ligation and puncture (CLP) in mice

**DOI:** 10.1186/s12931-020-01553-3

**Published:** 2020-11-04

**Authors:** Liu Yang, Sijie Liu, Silu Han, Yuhan Hu, Zhipeng Wu, Xiaoqian Shi, Baosen Pang, Yingmin Ma, Jiawei Jin

**Affiliations:** 1 Department of Respiratory and Critical Care Medicine, Beijing Chaoyang Hospital, Capital Medical University, No. 5 Jingyuan road, Beijing Chaoyang Hospital Jingxi Branch, Beijing, China; 2 Beijing Institute of Respiratory Medicine, Beijing, China; 3 The Clinical Research Center, Beijing Chaoyang Hospital, Capital Medical University, Beijing, China

**Keywords:** ARDS, Sepsis, HDL dysfunction, Pulmonary vascular endothelial cell, ALI

## Abstract

**Background:**

Septic-acute respiratory distress syndrome (ARDS), characterized by the acute lung injury (ALI) secondary to aberrant systemic inflammatory response, has high morbidity and mortality. Despite increased understanding of ALI pathogenesis, the therapies to prevent lung dysfunction underlying systemic inflammatory disorder remain elusive. The high density lipoprotein (HDL) has critical protective effects in sepsis and its dysfunction has a manifested contribution to septic organ failure. However, the adverse changes in HDL composition and function in septic-ARDS patients are large unknown.

**Methods:**

To investigate HDL remodeling in septic-ARDS, we analyzed the changes of HDL composition from 40 patients with septic-ARDS (A-HDL) and 40 matched normal controls (N-HDL). To determine the deleterious functional remodeling of HDL, A-HDL or N-HDL was administrated to C57BL/6 and apoA-I knock-out (KO) mice after cecal ligation and puncture (CLP) procedure. Mouse lung microvascular endothelial cells (MLECs) were further treated by these HDLs to investigate whether the adverse effects of A-HDL were associated with endothelial dysfunction.

**Results:**

Septic-ARDS patients showed significant changes of HDL composition, accompanied with significantly decreased HDL-C. We further indicated that A-HDL treatment aggravated CLP induced ALI. Intriguingly, these deleterious effects of A-HDL were associated with pulmonary endothelial dysfunction, rather than the increased plasma lipopolysaccharide (LPS). Further in vitro results demonstrated the direct effects of A-HDL on MLECs, including increased endothelial permeability, enhanced expressions of adhesion proteins and pro-inflammatory cytokines via activating NF-κB signaling and decreased junction protein expression.

**Conclusions:**

Our results depicted the remodeling of HDL composition in sepsis, which predisposes lung to ARDS via inducing ECs dysfunction. These results also demonstrated the importance of circulating HDL in regulating alveolar homeostasis.

## Background

Acute respiratory distress syndrome (ARDS) is presented as noncardiogenic pulmonary edema-induced hypoxia caused by acute lung injury (ALI) secondary to lung excessive inflammation [1]. An approximately 75% of ARDS is associated with sepsis and presents severe mortality and morbidity [2]. Owing to the vast surface area of pulmonary microvascular endothelium for effective gas exchange, the pulmonary vascular endothelial cells (ECs) are vulnerable to circulating stimuli during sepsis [3]. The pro-inflammatory mediators in circulation lead to ECs dysregulation with abnormal increases in the expression of pro-inflammatory cytokines and cellular adhesion proteins including vascular cell adhesion molecule 1 (VCAM-1), intercellular adhesion molecule-1 (ICAM-1) and E-selectins [4–6]. Excessive inflammation further impairs pulmonary microvascular integrity due to the decrease in endothelial cell–cell junction proteins (e.g. cadherin) and ECs apoptosis, resulting in pulmonary vascular permeability disruption, alveolar edema, additional immunocytes trafficking and uncontrolled alveolar inflammation [7]. Hence, inflammation-mediated pulmonary endothelial dysfunction is considered to be the main pathogenesis of septic-ARDS. Understanding the mechanism of circulating inflammatory imbalance in pulmonary endothelial dysfunction is of crucial importance.

Compared to other lipoproteins, HDL has a critical role in maintaining the endothelial integrity due to its anti-inflammatory and anti-oxidative properties [8]. Upon septic stresses, HDL processes the anti-inflammatory function through both neutralizing lipopolysaccharide (LPS) and alleviating ECs inflammatory responses [9]. Septic patients exhibit a marked reduction in plasma HDL cholesterol (HDL-C) level and the low level of HDL-C is a poor prognostic factor for severe sepsis [10–12]. In addition, an adverse transition of HDL to pro-inflammation was observed during acute inflammatory disorder diseases including sepsis [13–15]. Previous studies indicated that the abnormal oxidation of apoA-I by the myeloperoxidase (MPO) could be one of potential causes for the adverse transition of HDL in acute phase [13, 16]. However, the adverse remodeling of HDL in pathogenesis of septic-ARDS is large unknown due to the complexity of HDL components. It is therefore necessary to investigate the changes in HDL composition and function in septic-ARDS patients.

In this study, we, for the first time, examined the HDLs isolated from 40 septic ARDS patients (A-HDL) and matched 40 normal controls (N-HDL) through in vivo and *in intro* studies. In addition to the decrease in the plasma levels of HDL-C and HDL-apolipoproteins, we found significant alterations of HDL composition in ARDS patients. The further in vivo studies indicated that the treatment of A-HDL exacerbated CLP-induced mouse ALI, suggesting an adverse functional transition of A-HDL during septic-ARDS. The deleterious effects of A-HDL were associated with pulmonary endothelial deregulation, including increased endothelial permeability, aberrant expressions of cytokines and cellular adhesion proteins. These findings clearly revealed that the remodeling in HDL composition promotes septic-ARDS via endothelial dysfunction.

## Methods

### Subjects

All human studies were approved by the Ethics Committee of Beijing Chaoyang Hospital, Capital Medical University (Certificate No. 2018-KE-324) and the informed consents were signed by all subjects or their representatives. The cohort study enrolled adult ARDS subjects (aged from 18–85 years old) secondary to sepsis with the requirement of invasive mechanical ventilation (IMV) and without metabolic disorders including diabetes and hyperlipidemia from the medical ICU and paired adult healthy subjects (volunteers) without chronic and acute diseases from the health screening center at the Beijing Chaoyang Hospital, Capital Medical University. Septic-ARDS was defined in accordance with the Berlin definition and 2016 Sepsis 3.0 definition and the blood samples were collected within 24 h after ARDS confirmation. In total, the blood samples from 40 ARDS patients and 40 age and sex-matched healthy controls were collected (Table 1 for the clinical characteristics). We collected 12 ml of blood from ARDS patients and 6 ml of blood from control subjects, respectively. Blood was drawn into a 6-ml EDTA vacutainer and centrifuged at 3000 rpm for 10 min and the plasma was frozen at − 80 °C for further studies.

**Table 1:** Clinical and demographic characteristics of ARDS patients and normal subjects

Characteristics	ARDS patients (n = 40)	Healthy controls (n = 40)	P
Male, n (%)^a^	28 (70.0)	25 (62.5)	0.48
Age (years) ^b^	67 (55–74)	62 (57–64)	0.13
Etiology, n (%)		N/A	
Pneumonia	25 (62.5)		
Abdominal infection	15 (37.5)		
Underlying diseases, n (%)		N/A	
Hypertension	18 (45.0)		
Chronic respiratory disease	2 (5.0)		
PaO2/FiO2 ratio, n (%)		N/A	
200–300	8 (20.0)		
100–200	20 (50.0)		
≦100	12 (30.0)		
CRP (mg/dl)	12.0 (8.7–17.1)	N/A	
PCT (ng/ml)	2.7(0.7–18.4)	N/A	
APACHE II score	15 (11–24)	N/A	
SOFA score	7 (4–10)	N/A	
Complications, n (%)		N/A	
Liver disfunction	20 (50.0)		
Acute kidney injury	7 (17.5)		
Treatment during ICU, n (%)		N/A	
Vasopressor	24 (60.0)		
Parenteral nutrition	18 (45.0)		
Sedative	31 (77.5)		
IMV days	16 (9–32)	N/A	
28-day mortality, n (%)	13 (32.5)	N/A	

*PaO2* arterial oxygen tension, *FiO2* fraction of inspired oxygen, *CRP* C-reactive protein, *PCT* procalcitonin, *APACHE II*, Acute Physiology and Chronic Health Evaluation II, *SOFA* sequential organ failure assessment, *IMV* invasive mechanical ventilation

^a^Chi-square test

^b^Mann–Whitney U test

### Isolation of HDL subfractions

HDL (1.063 < density < 1.21 g/ml) was isolated from plasma by discontinuous density-gradient ultracentrifugation [17]. Plasma from 5 individuals with similar age and clinical situations (including etiology, APACHE II score and inflammatory condition) were pooled to improve the quality of isolated HDL. Mixed plasma samples were adjusted to a density of 1.3 g/ml with KBr and the plasma was carefully overlaid with normal saline after loaded to centrifuge tube. The samples were centrifuged at 350,000 g for 5 h at 4 °C and HDLs in the middle of the tubes were carefully collected by penetrating with a syringe. The lipoprotein fractions were then dialyzed against endotoxin-free phosphatebuffered saline (10 mM, PH7.4) at 4 °C for 24 h. HDLs were sterilized with 0.22 μm filter. The purity of HDLs were confirmed by the 10% SDS-PAGE electrophoresis. The concentration of HDLs were quantified through the measurement of apoA-I content by nephelometry.

### Cecal ligation and puncture (CLP) septic mouse model

The animals were bred at the animal facility of Institute of Genetics and Developmental Biology, Chinese Academy of Sciences. All animal procedures were approved by the Animal Care and Ethics Committee of the Institute of Genetics and Developmental Biology, Chinese Academy of Sciences and were performed in accordance with the Guide for the Care and Use of Laboratory Animals of the Chinese Academy of Sciences.

ApoA-I knockout (KO) mice on C57BL/6 background were obtained from The Jackson Laboratory. CLP was performed on 10-week-old mice. Briefly, mice were anesthetized by 2% sodium pentobarbital (110 mg/kg) and a 1.0–2.0 cm of midline incision was made below the diaphragm on shaved and sterilized abdomen (scrubbed with hair cream and povidone-iodine) to expose the cecum. Following a 30% ligation (light CLP) or a 50% ligation (moderate CLP), cecum was punctured twice with a 18-gauge needle and gently compressed to extrude a small amount of cecal material. The cecum was returned to the abdomen, and the muscle and skin incisions were closed with 4–0 silk suture. Sham group was similarly treated without ligation and puncture of the cecum. After the surgery, mice were resuscitated with 1 ml pre-warmed (37 ℃) phosphate-buffered saline subcutaneously. 24 h post CLP, the lung tissues were collected and subjected into further analyses.

### Cell experiments

Mouse lung microvascular endothelial cells (MLECs) were isolated from C57BL/6 mice. Briefly, the lung was perfused, lavaged and cut into small pieces which were in turn digested with the enzymes dispase and collagenase A (Sigma) for 60 min at 37 °C. Following digestion, single-cell suspensions were passed through a 70-μm filter to remove debris. Endothelial cells were isolated by positive selection using Microbeads binding to CD31. Flow cytometry confirmed that 90% of cells in the final suspension are CD31-positive. Primary MLECs were maintained in endothelium cell medium (Sciencell). For HDL treatment experiments, endothelial cells were cultured in endothelium cell medium (containing 1% FBS) with HDL (50 μg/ml) or human albumins (sigma).

### In vitro permeability assay

MLECs were cultured on transwell inserts (diameter: 6.5 mm, pore size: 0.4 μm, Corning). Until cells formed a monolayer, the culture medium in upper and lower compartments was changed to medium (1% FBS) with HDL (50 μg/ml) or human albumin and cells were cultured for 24 h. The fluorescent tracer, fluorescein isothiocyanate (FITC)-dextran (500 μg/ml, Sigma-Aldrich) was then added to the upper compartment. The permeability was determined by the diffused amount of FITC-dextran into the lower compartment measured by fluorescence intensity of the medium at 518 nm with excitation at 492 nm.

### Oxidative modification of apoA-I

The protein bands were digested according to an in-gel digestion procedure and were subjected into liquid chromatography–mass spectrometry (LC–MS/MS) analysis performed on an LTQ linear ion trap mass spectrometer. Modification analysis was carried out by LC–MS/MS searching the data specifically against the sequence of apoA-I using the program Sequest bundled into Proteome Discoverer 1.3 (Thermo Scientific, San Jose, CA, USA). The native reference peptide (NRP) method was used for the quantification of modified peptides present in a tryptic digest by utilizing an unmodified peptide from the protein of interest that is also formed in the tryptic digestion as the internal standard. The peak areas for interesting peptides were determined and normalized to the peak areas of the reference peptides as an index of the relative amounts of these peptides [18].

### Lung lavage analyses

The bronchoalveolar lavage fluid (BALF) was collected as described previously [19, 20]. Briefly, 1 ml saline was instilled into lung through a 20-gauge blunt-tipped needle inserted into the trachea and aspirated three times. The supernatant of BALF (400 g × 10 min) was used for the measurements of total BALF protein (Bradford) and inflammatory cytokines by Elisa kits.

### Histological analyses

Lung dissected out from mice subjected into designed procedure were fixed in 4% PFA and embedded in paraffin. These paraffin embedded tissues were sliced into 5 μm-thick sections for Hematoxylin and eosin (HE) (ScyTek Laboratories). Immnunohistochemistry (IHC) was performed as a standing protocol and the antibody used was VCAM1 (CST, 39,036).

The histopathologic degree of lung injury was evaluated through a double-blind examination and scored semi-quantitatively by a scale of 0 to 4 (0, appears normal; 1, light; 2, moderate; 3, strong; 4, intense) for edema, inflammation, hemorrhage and area of structural impairment, based on 10 fields of lung parenchyma (200 × magnification). A mean score for each of the parameters was then calculated. The final lung injury score was obtained by averaging the score from the animals within each group.

### Wet-to-dry lung weight ratio and Evans Blue dye leakage

The right up lobe of lung was collected to assess the lung wet-to-dry weight ratio by the gravimetric method. After the wet lung weight was measured, the lung was incubated at 60 °C for 72 h. Then, the dry lung weight was measured and the ratio of wet-to-dry weight was calculated.

To examine the alveolar microvascular leakage after CLP, mice were injected via the tail vein with Evans Blue dye (50 mg/kg). Three hours after dye injection, BALF was collected and the supernatant of remaining BALF (400*g* × 10 min) was used for the measurements of absorbance at 620 nm.

### The Levels of Cytokines and apolipoproteins

The HDL components including apolipoproteins, serum amyloid A (SAA), paraoxonase-1 (PON1) and myeloperoxidase (MPO) were quantified by Quantitative Competitive Elisa kits from Hermes Criterion Biotechnology. The plasma levels of human apolipoproteins, PON1, MPO and mouse HDL were measured by Elisa kits from Cloud-clone. The levels of all cytokines were measured by Elisa kits from R&D Systems.

### Quantitative Real-time PCR

Total RNA was extracted from mouse lungs and MLECs using TRIzol® Reagent (Invitrogen) according to the manufacturer’s manual. RNA was transcribed into cDNA using random oligonucleotide hexamers in First Strand cDNA Synthesis Kit (Invitrogen). The data were normalized to the RPL19 content and analyzed by the 2^−△△^Ct method relative to control groups. The primers used in qPCR: TNF-α: CAGGCGGTGCCTATGTCTC, CGATCACCCCGAAGTTCAGTAG; IL-1β: GAAATGCCACCTTTTGACAGTG, TGGATGCTCTCATCAGGACAG; MCP-1: TAAAAACCTGGATCGGAACCAAA, GCATTAGCTTCAGATTTACGGGT; IL-6: CTGCAAGAGACTTCCATCCAG, AGTGGTATAGACAGGTCTGTTGG.

### Immunoblot analyses

Protein extracts prepared from tissues and cells were subjected to immunoblot analyses with antibodies against: VE-Cadherin from Thermo Fisher, VCAM1 and P-p65 from CST, ICAM1 form Abcam, P65 and GAPDH form Santa Cruz.

### Statistical analysis

Data are presented as n for categorical variables, mean ± SEM (stand error of the mean) for normally distributed continuous variables, medians (25th–75th percentiles) for no-normally distributed continuous variables. To compare continuous variables, the Shapiro–Wilk test was used to test the normality of the data. Statistical comparisons between 2 groups were performed using χ2 test for categorical variables, the Mann–Whitney U test for non-normally distributed continuous variables and the two-tailed Student’s t-test for normally distributed continuous variables. Comparisons among groups were performed using 1-way or 2-way ANOVA followed by post hoc multiple comparison tests where appropriate. A p value of < 0.05 indicated significant difference between groups.

## Results

### The dysregulation of HDL in ARDS patients

To determine the changes of HDL in septic-ARDS, the plasma samples were collected from 40 ARDS patients and 40 matched healthy controls (Table 1 for the clinical characteristics). Compared to healthy controls, ARDS patients showed significantly decreased plasma levels of HDL-C and HDL-apolipoproteins (apoA-I, apoA-II and apoA-IV, apoC-III) and the increased level of apoE. These patients also exhibited the remarkable increases of inflammatory indexes (CRP and PCT), while the reduction of paraoxonase-1 (PON1) was observed in these patients (Table 2). No significant difference in HDL-C level was found among mild, medium and severe ARDS patients (data not shown).

**Table 2:** The plasma levels of HDL-C and key HDL-related proteins

	ARDS patients (n = 40)	Healthy controls (n = 40)	P
HDL-C, mmol/L^b^	0.55 (0.40–0.88)	1.46 (1.15–1.92)	< 0.0001
apoA-I, μg/ml^b^	35.6 (24.0–43.0)	88.5 (70.3–111.7)	< 0.0001
apoA-II, μg/ml^b^	32.0 (22.3–45.9)	58.0 (48.3–70.9)	< 0.0001
apoA-IV, μg/ml^b^	7.5 (3.8–13.3)	28.7 (24.1–35.5)	< 0.0001
apoC-III, μg/ml^b^	22.6 (21.1–25.0)	25.9 (23.7–28.7)	< 0.0001
apoE, μg/ml^a^	28.5 ± 1.0	20.6 ± 0.8	< 0.0001
MPO, μg/ml^b^	0.6 (0.3–1.0)	0.7 (0.4–1.0)	0.61
PON1, ng/ml^b^	5.5 (4.2–7.1)	7.0 (6.1–9.1)	< 0.0001
MPO/PON1^b^	105.8 (41.1–193.8)	91.9 (58.8–129.6)	0.28

^a^Student's-t test, ^b^Mann–Whitney U test. *HDL-C* high density lipoprotein-cholesterol, *apo* apolipoprotein, *MPO* myeloperoxidase, *PON1* paraoxonase-1

**Fig. 1:**
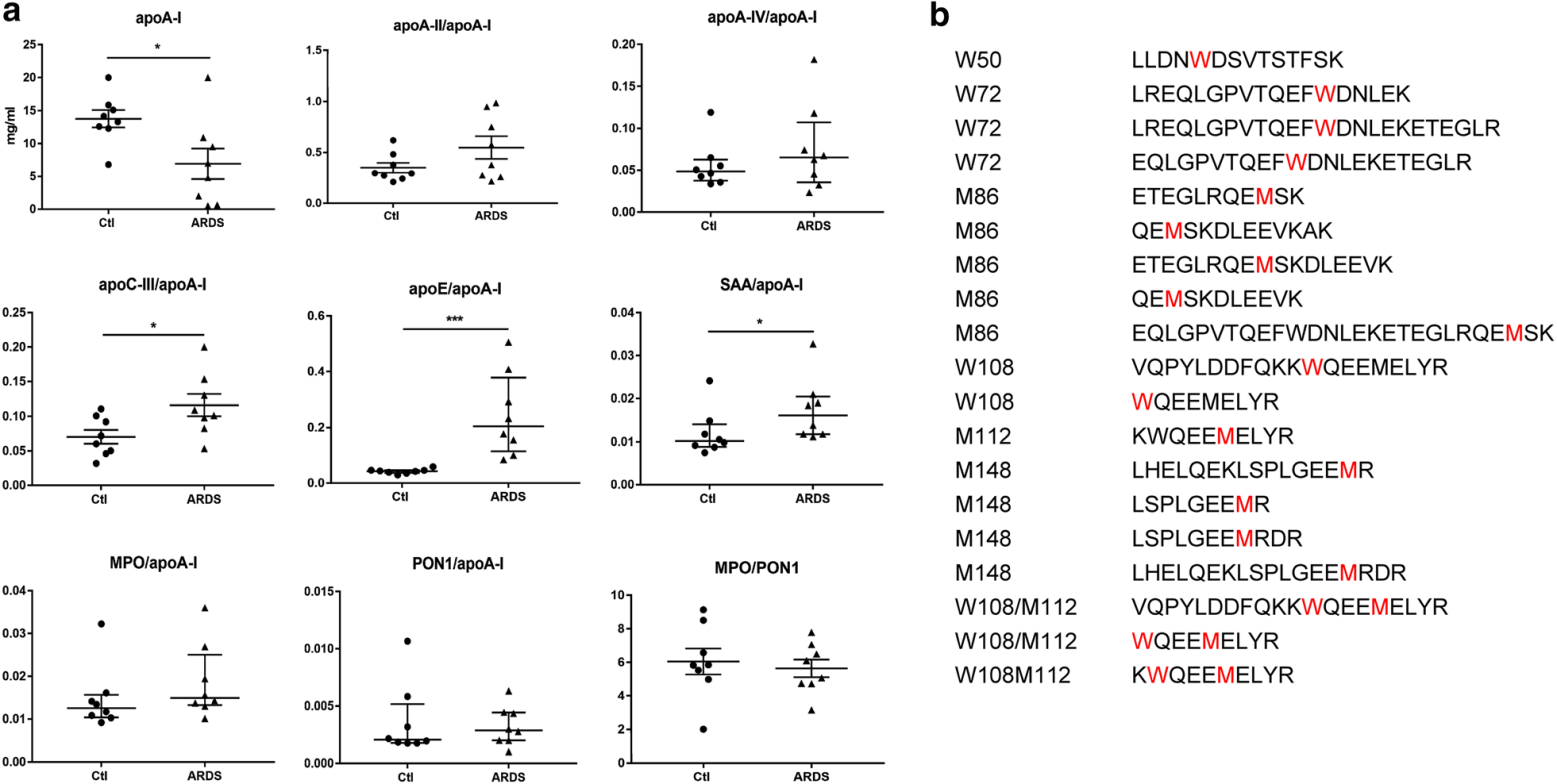
The alteration of HDL components in ARDS patients. The plasma samples from 40 ARDS patients and 40 healthy controls were subjected into HDL isolation and further assays. **a** The components in HDLs isolated from ARDS patients and control subjects are measured and the constituents are presented as the ratio to apoA-I. (n = 8 per group, 1 HDL sample isolated from 5 subjects). **b** The LC–MS/MS analysis show the same patterns of oxidative modification sites (amino acid marked with red color) in apoA-I from ARDS patients and control subjects (4 HDL samples per group, 1 HDL sample from 5 subjects). *p < 0.05 and ***p < 0.001 versus controls. Ctl: control subjects, PON1: paraoxonase-1, MPO: myeloperoxidase

Following HDL isolation, the amount of important protein components were measured and the ratios to apoA-I were calculated to determine HDL composition. A plasma mixture from 5 patients with similar age and clinical situations was used for the isolation procedure to improve the quality. Compared to N-HDL, A-HDL displayed a significant decrease in apoA-I. The ratios of apoE, apoC-III and SAA to apoA-I increased in A-HDL, while the fractions of MPO, PON1, apoA-II and apoA-IV were comparable between N-HDL and A-HDL (Fig. 1a). Additionally, since deregulated apoA-I oxidation accounted for HDL dysfunction, we compared the oxidative modifications of apoA-I in N-HDL and A-HDL by LC–MS/MS (4 HDL samples per group, 1 HDL sample isolated from 5 subjects pooled plasma). However, both groups exhibited the same patterns of oxidative sites (Fig. 1b) and failed to show any significant difference in the oxidation levels of each site (data not shown).

### The plasma HDL from ARDS patients promotes CLP-induced ALI

Owing to the alterations in HDL composition observed in septic-ARDS patients, we further investigated the functional changes of A-HDL, by administrating A-HDL or N-HDL to C57BL/6 mice through tail vein (50 mg/kg, PBS as control), immediately after moderate CLP surgery. In order to exclude the potential deleterious effects due to the inflammatory cytokines contaminated in HDLs, we measured the levels of inflammatory cytokines (TNF-a and IL-8) in plasma and isolated HDLs. As expected, the levels of TNF-a and IL-8 in plasma from ARDS patients were significantly higher than these in control subjects, while there were no statistic differences in levels of TNF-a and IL-8 between the A-HDL and N-HDL (Additional file 1: Figure S1A).

**Fig. 2:**
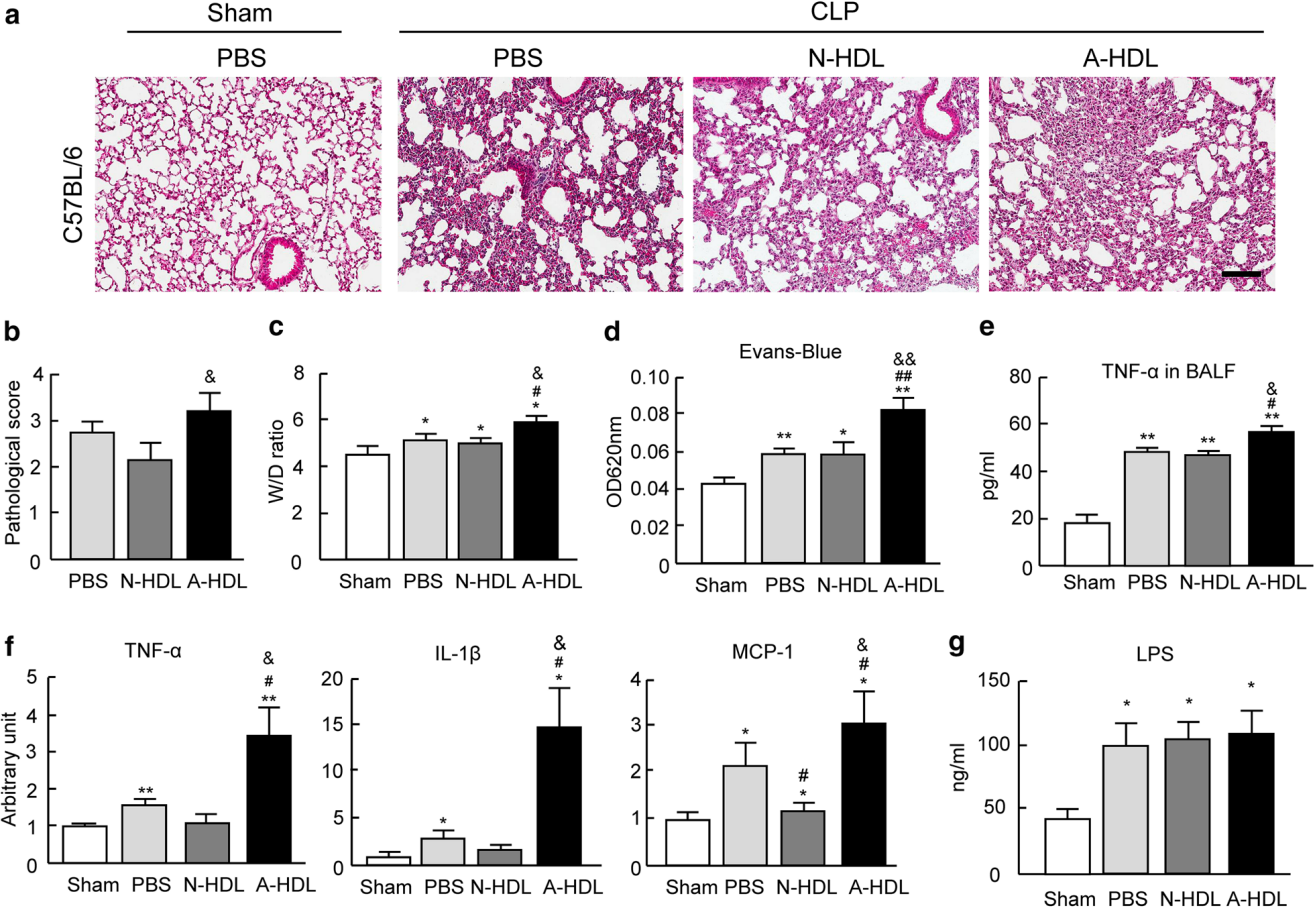
The plasma HDL from ARDS patients promotes CLP-induced ALI in C57BL/6 mice. C57BL/6 mice were treated with PBS, N-HDL or A-HDL after moderate CLP (50% ligation). **a** Representative hematoxylin and eosin–stained lung sections. **b** The degree of lung injury was scored by a scale of 0 to 4 according to edema, inflammation, hemorrhage and the area of structural impairment (n = 7 per group). The ratios of lung wet/dry weight (**c**) and the Evans Blue leakage assay (**d**) (n = 5–8 per group). **e** The level of TNF-α in BALF (n = 5 per group). **f** The mRNA expressions of pro-inflammatory cytokines (TNF-a, IL-1 and MCP1) in lung tissues by qPCR (n = 4–5 per group). **g** The level of plasma LPS (n = 5–8 per group). *p < 0.05 and **p < 0.01 versus sham group; ^#^p < 0.05, ^##^p < 0.01 versus PBS treatment group; ^&^p < 0.05 and ^&&^p < 0.01 versus N-HDL treatment group. CLP: Cecal ligation and puncture, N-HDL: HDL from normal subjects, A-HDL: HDL from ARDS patients. Scale bar: 100 μm

Although HDL treatments failed to cause obvious lung histopathologic changes and inflammation on sham mice without CLP (Additional file 1: Figure S1B), the administration of A-HDL, but not N-HDL, significantly promoted CLP-induced ALI indicated by severe alveolar histopathologic disruption including thickening alveolar septum, inflammatory cells infiltration, patchy hemorrhage areas (Fig. 2a, b). A-HDL treatment also caused severe lung edema indicated by the markedly increased ratio of lung wet/dry weight (Fig. 2c). The Evans Blue leakage assay further indicated significantly aggravated pulmonary endothelial permeability by A-HDL treatment 4 h after CLP (Fig. 2d).

The severe ALI in A-HDL treated mice was coupled with an exaggerated inflammatory response determined by the increased levels of TNF-α in BALF and the marked upregulation of TNF-α, IL-1β and MCP1 in the lung (Fig. 2e, f). Intriguingly, no difference was observed in the plasma level of LPS between mice treated by A-HDL and N-HDL, suggesting that the enhanced ALI by A-HDL was not due to abnormal increase in plasma LPS (Fig. 2g).

**Fig. 3:**
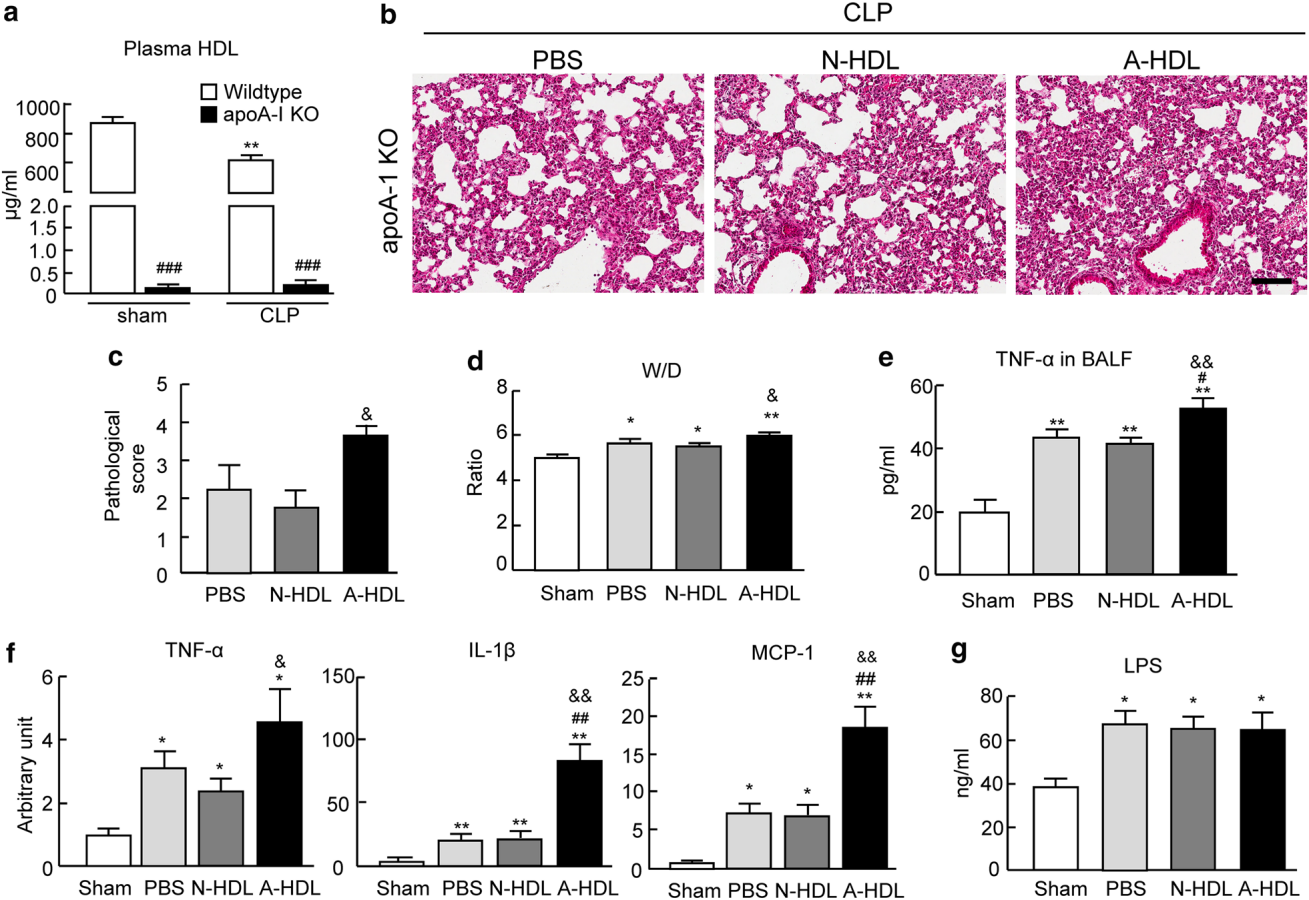
The plasma HDL from ARDS patients promotes CLP-induced ALI in apoA-I KO mice with the deficiency of endogenous HDL. **a** A depleted level of plasma HDL is observed in apoA-I KO mice and the moderate CLP surgery caused a marked decrease in the level of plasma HDL in WT mice (n = 5–8 per group). **b** Representative hematoxylin and eosin–stained lung sections from apoA-I KO mice treated with PBS, N-HDL or A-HDL after light CLP. **c** The degree of lung injury (n = 7 per group). **d** The ratio of lung wet/dry weight (n = 5 per group). **e** The level of TNF-α in BALF after CLP (n = 5 per group). **f** The mRNA expressions of pro-inflammatory cytokines (TNF-a, IL-1 and MCP1) in lung tissues by qPCR analyses (n = 5 per group). **g** The level of plasma LPS after CLP surgery (n = 5–8 per group). **p < 0.01 versus sham group of WT mice and ^####^p < 0.0001 versus sham group in **a**. *p < 0.05 and **p < 0.01 versus sham group; ^#^p < 0.05, ^##^p < 0.01 versus PBS treatment group; ^&^p < 0.05 and ^&&^p < 0.01 versus N-HDL treatment group in **c** to **g**. CLP: Cecal ligation and puncture, N-HDL: HDL from normal subjects, A-HDL: HDL from ARDS patients. Scale bar: 100 μm

Given the potential effects of endogenous mouse HDL in these in vivo studies, the HDLs were administrated into apoA-I KO mice which showed massive depleted plasma HDL (Fig. 3a). These KO mice displayed severe ALI induced by moderate CLP with exacerbated pulmonary inflammation (Additional file 2: Figure S2). Since moderate CLP caused high mortality on KO mice, these mice were subjected into light CLP procedure followed by HDL administration. In line with observations in C57BL/6 mice, A-HDL treatment enhanced ALI/ARDS phenotypes in apoA-I KO mice after CLP including alveolar histopathologic changes, lung permeability, lung edema and alveolar inflammation (Fig. 3b–f), while A-HDL and N-HDL treated mice showed the comparable levels of plasma LPS in these mice (Fig. 3g). These results further clearly confirmed that the adverse remodeling of HDL facilitates sepsis-induced ALI/ARDS and these deleterious effects are not due to the abnormal capability of LPS neutralization.

### A-HDL remodeling promotes CLP-induced dysfunction of pulmonary endothelium

**Fig. 4:**
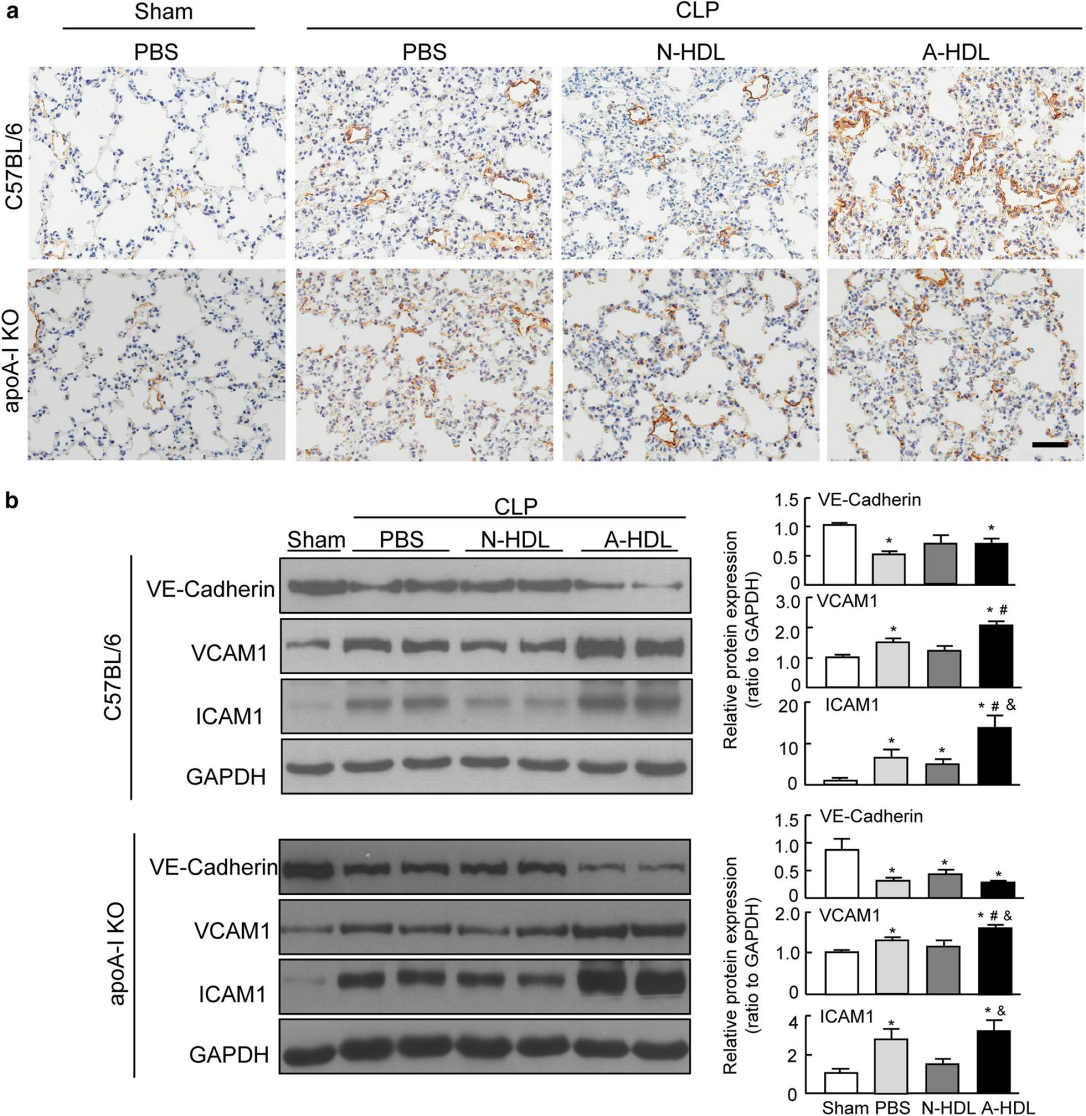
A-HDL remodeling promotes CLP-induced deregulation of pulmonary endothelium. **a** Representative immunohistochemistry of VCAM1 on lung sections from C57BL/6 and apoA-I KO mice treated with PBS, N-HDL or A-HDL after CLP. **b** Immunoblot analyses and the ratio of densitometric measurement to GAPDH are represented by the bar graphs (n = 3–5 per group). *p < 0.05 versus sham group; ^#^p < 0.05 versus PBS treatment group; ^&^p < 0.05 versus N-HDL treatment group. CLP: Cecal ligation and puncture, VCAM1: vascular cell adhesion molecule-1, ICAM1: intercellular adhesion molecules-1, N-HDL: the HDL from normal subjects, A-HDL: the HDL from ARDS patients. Scale bar: 100 μm

To determine whether deleterious effects of A-HDL could be associated with pulmonary endothelial deregulation, we examined the adhesion proteins involved in endothelial cell–cell junction and leukocyte recruitment. CLP surgery caused significant increases in VCAM1 and ICAM1 and decrease in VE-cadherin in the lungs, whereas A-HDL treatment caused exacerbated changes suggesting a worse deregulation of pulmonary vascular endothelium (Fig. 4a, b). These findings were consistent with severe ALI/ARDS phenotype observed in these mice, suggesting that the adverse remodeling in HDL is associated with the dysfunction of pulmonary endothelium during the development of ARDS.

### HDL from ARDS patients promotes the dysfunction of primary cultured pulmonary microvascular endothelial cells

**Fig. 5:**
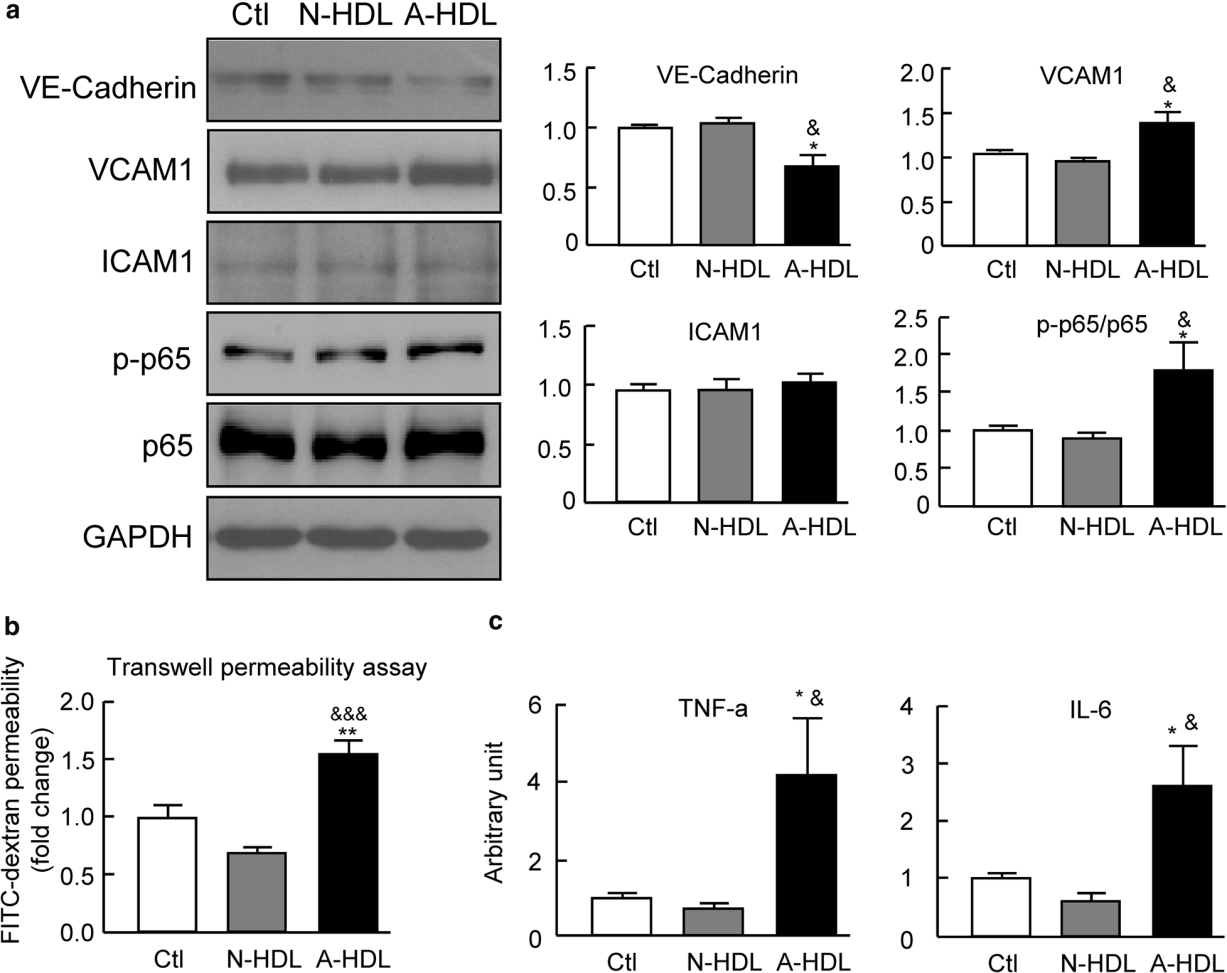
The plasma HDL from ARDS patients promotes the dysfunction of primary pulmonary microvascular endothelial cells. Mouse lung microvascular endothelial cells (MLECs) were treated with N-HDL, A-HDL and PBS with human albumins as control (50 μg/ml, 24 h). Western blot analysis for junctional protein (VE-cadherin), vascular adhesion markers (VCAM1 and ICAM1) and Phospho-NF-κB p65. (n = 4 per group). **b** The monolayers of MLECs on transwell inserts were treated with HDLs (50 μg/ml, 24 h) and the permeability was determined by the diffusion of tracer (FITC-dextran) into the lower compartment. The permeability change was presented as the fold change of fluorescence intensity relative to controls (n = 4 per group). **c** qPCR analyses of the mRNA expressions of cytokines (TNF-a and IL-6) in MLECs treated with HDLs (50 μg/ml, 12 h). (n = 5 per group). *p < 0.05 and **p < 0.01 versus control group; ^&^p < 0.05 and ^&&&^p < 0.001 versus N-HDL treatment group. *VCAM1* vascular cell adhesion molecule-1, *ICAM1* intercellular adhesion molecules-1, *Ctl* control, *N-HDL* HDL from normal subjects, *A-HDL* HDL from ARDS patients

Since A-HDL and N-HDL treated mice show similar plasma LPS level, we reasoned that the A-HDL might have direct deleterious effects on lung vascular endothelial cells to render the lung more susceptible to sepsis-induced endothelial dysfunction. To examine this hypothesis, isolated MLECs (CD31-positive, Additional file 1: Figure S1C) were exposed to medium containing N-HDL, A-HDL or PBS with human albumins as control. The cells treated with A-HDL showed marked reduction of VE-cadherin and induction of VCAM1, although A-HDL treatment failed to increase ICAM1 expression (Fig. 5a). Of note, accompanied with VE-cadherin reduction, A-HDL treatment caused significant increase in endothelial permeability, determined by Transwell permeability assay on the diffusion of FITC-dextran tracer (Fig. 5b).

Additionally, A-HDL exposure also caused marked increased expression of pro-inflammatory cytokines including TNF-α and IL-6 (Fig. 5c). Since the activation of NF-κB signaling is associated with the induction of inflammatory cytokines, the change in P-p65/p65 NF-κB was then measured. A-HDL treated cells exhibited a high ratio of P-p65/p65, whereas N-HDL exposure failed to cause activation of p65, suggesting a direct effect of A-HDL on the activation of pro-inflammatory signaling (Fig. 5a). These findings suggested that the dysfunction of HDL may predispose the lung to sepsis-induced ALI/ARDS through the direct deleterious effects on endothelial cells.

## Discussion

Herein, we indicated that sepsis-induced changes of HDL quality predispose the lung to ALI/ARDS via exacerbating pulmonary endothelial dysfunction, evidenced by key findings: (1) The septic-ARDS patients with increased pro-inflammatory cytokines showed marked alterations of HDL composition including the fractions of apolipoproteins and SAA. (2) The HDL from septic-ARDS patients showed deleterious remodeling to exacerbate CLP-induced ALI without increasing the plasma level of LPS. (3) The remodeling of HDL caused direct adverse effects on pulmonary vascular endothelial cells via enhanced pro-inflammatory properties. These findings advance the pathogenesis and therapeutic perspectives of septic-ARDS.

### The remodeling of HDL in ARDS patients

Since apoA-I as the major apolipoprotein in HDL mediates critical protective functions of HDL including LPS neutralization and reversal cholesterol transport (RCT) from macrophages, the dysfunction of apoA-I has a critical contribution to inflammation-associated acute and chronic pulmonary diseases [21–23]. However, apoA-I can be released in alveoli by alveolar epithelial cells and macrophages to regulate lipid homeostasis and inflammation [22–25]. Therefore, the observations of apoA-I dysfunction might not fully represent the functional remodeling of HDL in septic-ARDS with systemic inflammatory disorder.

Our studies showed the significant decreases in plasma levels of HDL-C and HDL-associated apolipoproteins with marked alterations in HDL composition in these patients. These observations suggest that the depletion of HDL is likely associated with the development of septic-ARDS, although the correlation between HDL level and ARDS severity failed to reach statistic significance due to the limited number of patients. Our findings are consistent with the previous studies that patients with sepsis showed a lower level of HDL than patients with trauma in ICU. These studies also indicated that the decreased level of HDL-C was an independent predictor for persistent organ failure and mortality in acute pancreatitis [26–28].

In addition, excessive inflammatory deregulation also caused changes in HDL profile indicated by proteomics studies on the HDL from acute coronary syndromes (ACS) patients showed increases in apoA-IV and apoE, suggesting a critical role of HDL remodeling in acute diseases [29, 30]. HDL isolated from uremia patients contains enriched components of apoC-III, SAA and triglycerides, which have detrimental effects on HDL function such as RCT from macrophages [31]. In line with these observations, our data indicated the changes of apolipoprotein fractions in the HDL from septic-ARDS patients, including significant increases in apoC-III and apoE. Notably, we also observed a marked increase in the fraction of SAA, an acute-phase response protein associated with enhanced inflammation. Acute inflammation stress causes an increase of SAA fraction in HDL via displacing HDL-associated proteins (apoA-I and PON1) by circulating SAA on the HDL surface [32, 33]. Consistently, we also found that the increase in SAA fraction was accompanied by a significant decrease in apoA-I faction in A-HDL, suggesting that the replacement of apoA-I by SAA could contribute the adverse transition of A-HDL.

The anti-oxidative and anti-inflammatory functions of HDL is largely attributed to the functions of apoA-I and PON1. ApoA-I is susceptible to oxidation mediated by MPO and such modification impairs its anti-inflammatory function [16]. However, our analyses failed to find significant increase in oxidative level of apoA-I in A-HDL. Furthermore, MPO and PON1, as HDL-associated proteins, bind and interact with HDL by forming a complex, wherein PON1 inhibits MPO activity, while MPO inactivates PON1 [34]. In line with similar oxidative level of apoA-I, there are no significant differences in MPO/PON1 ratio between A-HDL and N-HDL. Taken together, these observations suggest that remodeling of A-HDL is likely associated with the development of ARDS and the profound increase of SAA might have large contribution to adverse functional change of HDL.

### The remodeling of HDL predispose lung to ARDS via promoting disruption of pulmonary vascular endothelial homoeostasis

The vast surface area of pulmonary microvascular endothelium for effective gas exchange makes ECs vulnerable to circulating stimuli, especially upon infectional or sterile inflammatory disorders [3]. The disruption of pulmonary endothelial homoeostasis therefore plays a causative role for sepsis-induced ARDS [35]. In our studies, A-HDL exposure promoted CLP-induced endothelial disruption indicated by increased lung permeability and severe alveolar inflammation, which is associated with the marked decrease of junctions protein VE-cadherin and the increase of intercellular adhesion proteins for alveolar leukocyte recruitment. These observations suggest that A-HDL aggravated endothelial dysfunction via both endothelial integrity disruption and endothelial inflammatory activation. Additionally, although the extrinsic endothelial cell apoptosis has been shown to be unregulated in ALI/ARDS [6], we failed to observe significantly enhanced apoptosis in the lung from A-HDL treated mice, suggesting that A-HDL exposure would promote the pro-inflammatory activation of endothelial cells rather than enhancing cell apoptosis.

Upon systemic inflammatory activation, circulating pro-inflammatory mediators activate pulmonary endothelial cells, characterized by increased expressions of pro-inflammatory cytokines and cell surface adhesion proteins [36, 37]. Herein, our in vitro studies showed that the exposure of A-HDL on primarily cultured MLECs caused marked inductions of TNF-α, IL-6 and VCAM1 as well as the reduction of VE-cadherin with increased cell permeability. These interesting findings, for the first time, provide direct evidence that the remodeling of HDL during septic-ARDS causes direct deleterious effects on pulmonary microvascular endothelial cells, suggesting the significance of HDL in crosstalk between pulmonary and systemic inflammatory regulation during ARDS. Such direct effects of HDL on endothelial cells are in line with findings in cardiovascular diseases studies showing that HDL regulates endothelial cell function via the interaction between HDL and endothelial cells [38]. However, the interaction and downstream regulation mechanisms in such acute lung injury-induced ARDS could be different from the findings in chronic cardiovascular diseases such as atherosclerosis. Therefore, it is worth to further investigate the mechanism involved in the interaction between HDL and pulmonary endothelial cells in ARDS.

## Conclusions

In conclusion, our results depicted a sepsis-induced remodeling both in HDL quantity and quality, which predisposes lung to ALI/ARDS via inducing pulmonary endothelial dysfunctions. These results, for the first, support that HDL remodeling, other than apoA-I modification, plays a critical role in the regulation of alveolar inflammation during acute systemic inflammatory disorder. These findings also advance the knowledge about the role of metabolic regulation in pathogenesis of septic-ARDS, which might share additional light to developing effective biomarkers for ARDS treatment.

## Supplementary information

**Additional file 1: Figure S1.** The inflammatory properties of HDL samples, their effects on C57BL/6 mice and the verification of isolated MLVECs. (A) The inflammatory cytokines TNF-a and IL-8 in plasma and HDLs from ARDS patients and control subjects. (n = 40 per group for plasma; n = 8 per group for HDLs, 1 HDL sample from 5 subjects), ****p < 0.0001 versus controls, ns: no significance, Ctl: control subjects. (B) Hematoxylin and eosin (HE) staining on lung sections from C57BL/6 mice and sham C57BL/6 mice treated by N-HDL, A-HDL and PBS. The treatments of N-HDL and A-HDL failed to cause obvious pathological changes. Scale bar: 100 pm. (C) The Immunofluorescence staining of CD31, a endothelial marker, on the primary cultured pulmonary vascular endothelial cells (MLECs) isolated from C57BL/6 mice.

**Additional file 2: Figure S2.** The depletion of HDL promotes CLP-induced ALI in mice. (A) Hematoxylin and eosin (HE) staining on lung sections indicated severe induced disruption of alveolar structures by moderate CLP. The degree of lung injury was scored (n = 7 per group). (B) The marked deterioration of lung vascular permeability in KO lungs was indicated by an increased ratio of lung wet/dry weight and the Evens Blue leakage assay (n = 4–6 per group). (C) qPCR analyses of the mRNA expressions of pro-inflammatory cytokines in lung tissues after CLP (n = 3–7 per group). (D) The levels of plasma LPS after CLP surgery (n = 3–7 per group).

## Data Availability

All data generated or analyzed during this study are included in this published article and its supplementary information files.

## Abbreviations

ARDS: Acute respiratory distress syndrome
ALI: Acute lung injury
ICU: Intensive care unit
HDL-C: High density lipoprotein-cholesterol
IMV: Invasive mechanical ventilation
Apo: Apolipoprotein
CLP: Cecal ligation and puncture
LPS: Lipopolysaccharide
MLECs: Microvascular endothelial cells
VCAM-1: Vascular cell adhesion molecule-1
ICAM-1: Intercellular adhesion molecule-1
MPO: Myeloperoxidase
PON1: Paraoxonase 1
KO: Knockout
LC–MS: Liquid chromatography–mass spectrometry
NRP: Native reference peptide
BALF: Bronchoalveolar lavage fluid
HE: Hematoxylin and eosin
IHC: Immnunohistochemistry
SAA: Serum amyloid A
RCT: Reversal cholesterol transport
ACS: Acute coronary syndrome
CRP: C-reactive protein
PCT: Procalcitonin
PaO2: Arterial oxygen tension
FiO2: Fraction of inspired oxygen
APACHE II: Acute physiology and chronic health evaluation II
SOFA: Sequential organ failure assessment
